# 同时性多原发肺癌与肺内转移鉴别方法研究进展

**DOI:** 10.3779/j.issn.1009-3419.2021.101.12

**Published:** 2021-05-20

**Authors:** 继凡 王, 特 张, 翰林 丁, 高超 董, 林 许, 峰 蒋

**Affiliations:** 210009 南京，南京医科大学附属肿瘤医院，江苏省肿瘤医院胸外科，江苏省肿瘤防治研究所，江苏省恶性肿瘤分子生物学及转化医学重点实验室 Department of Thoracic Surgery, the Affiliated Cancer Hospital of Nanjing Medical University, Jiangsu Cancer Hospital, Jiangsu Institute of Cancer Research; Jiangsu Key Laboratory of Molecular and Translation Cancer Research, Nanjing 210009, China

**Keywords:** 多原发性肺癌, 肺内转移, 肺肿瘤, 精准诊断, Multiple primary lung cancer, Intrapulmonary metastasis, Lung neoplasms, Accurate diagnosis

## Abstract

多原发性肺癌（multiple primary lung cancer, MPLC）是指在同一患者肺内不同部位同时或先后发生两个或两个以上原发病灶的肺癌，以诊断时间间隔6个月为界，MPLC分为同时性MPLC（synchronous MPLC, sMPLC）和异时性MPLC（metachronous MPLC, mMPLC）。sMPLC与肺内转移（intrapulmonary metastasis, IM）在治疗策略及预后上有着极大的差异。然而，目前临床上对于二者的区分鉴别存在许多争议。本文总结了目前诊断MPLC的主要方法，并重点介绍了区分MPLC和IM的最新研究进展，旨在为多灶性肺癌患者精准的诊断和治疗提供理论依据。

## Abstract (en)

Multiple primary lung cancer (MPLC) refers to lung cancer in which two or more primary lesions occurred simultaneously or successively in different parts of the same patient's lungs. The diagnosis interval is 6 months. MPLC is divided into synchronous MPLC (sMPLC) and metachronous MPLC (mMPLC). sMPLC and intrapulmonary metastasis (IM) are different in treatment strategies and prognosis. However, there are many controversies about the distinction between the two in clinical practice. This article summarizes the current main methods of diagnosing MPLC, and focuses on the latest research progress in distinguishing MPLC from IM. It aims to provide a theoretical basis for accurate diagnosis and treatment of patients with multifocal lung cancer.

据全球癌症流行病学数据GLOBOCAN 2020年的统计显示，肺癌的发病率和死亡率在全部恶性肿瘤中居于首位（全部人群）^[1]^。我国的肺癌发病率和死亡率也逐年上升，部分原因可能是低剂量计算机断层扫描（computed tomography, CT）的普及使得肺癌的检出率逐渐升高。多原发性肺癌（multiple primary lung cancer, MPLC）也越来越多的被人们发现和认识。MPLC是指在同一患者肺内不同部位同时或先后发生两个或两个以上原发病灶的肺癌，以诊断时间间隔6个月为界，MPLC分为同时性MPLC（synchronous multiple primary lung cancer, sMPLC）和异时性MPLC（metachronous multiple primary lung cancer, mMPLC）^[2, 3]^。在临床上，MPLC以腺癌多见，常需要与肺癌肺内转移（intrapulmonary metastasis, IM）相鉴别，二者的治疗方法也截然不同。研究^[4]^发现，MPLC和IM预后存在明显的差异。因此，准确地对MPLC尤其是sMPLC和IM进行鉴别显得尤为重要。然而，目前临床工作中对于二者的鉴别仍没有达成共识。本文主要从组织病理学、影像学和分子遗传学三个角度总结了近年来用来鉴别sMLPC和IM的方法。

## 1 组织病理学

组织病理学是诊断肺癌的金标准，但是当多灶性肺癌表现同一种组织学类型，尤其是腺癌时，其很难将sMPLC和IM进行有效的区分。Martini和Melamed^[2]^在1975年给出了MPLC的诊断标准并首次提出sMPLC和mMPLC的概念。sMPLC指两个肿瘤孤立，病理类型可能相同或不同。在病理相同的情况下，肿瘤一般分布在不同肺段、肺叶或不同侧肺，肿瘤原位起源且没有肺外转移或淋巴扩散的证据。Martini标准虽然缺乏区分sMLPC和IM的方法，但是仍具有重大临床意义。1995年Antakli等^[5]^对该标准进行了补充，增加了DNA倍体检测来区分MPLC和IM，但是由于技术及经济效益的制约，并未得到广泛应用。2003年，美国胸科医师协会（American College of Chest Physicians, ACCP）推荐了新的MPLC诊断标准^[6]^，并于2007年^[7]^和2013年^[8]^做出了更新。ACCP标准对于sMPLC诊断增加了分子遗传学的特征，特别强调了MPLC的诊断需要多学科联合，在一定程度上解决了MPLC和IM鉴别难题。

综合组织学评估（comprehensive histologic assessment, CHA）的方法在2009年由Girard等^[9]^提出。CHA通过更加详细地评估多个病变之间病理特征的差异来区分转移和多原发。其不仅评估了以10%为增量的组织学亚型的百分比，而且还评估了细胞学和基质特性。如果肿瘤对具有不同的组织学类型，或者癌症的主要亚型不同（即腺泡、乳头），或者当肿瘤对为鳞状细胞癌时，细胞和基质特征不同，则将肿瘤视为MPLC。如果肿瘤的组织学类型或细胞学和基质特征百分比相似，则认为他们是肿瘤内转移。该方法分类结果与分子分类的方法高度一致。在此基础上，研究发现将CHA和其他病理因素相结合可以进一步提高判断的准确性。Sun等^[10, 11]^通过CHA与低等级鳞状细胞成分或伴轻度核型异型的非黏液性鳞状细胞性病变相结合的方法来鉴别sMLPC和IM，与单纯的CHA方法相比，鉴别准确性有了明显提升。尽管这些方法提供了更准确的鉴别可能，但是它们仍然需要更大的样本去进一步证实。

总体来说，组织病理学的方法依旧是目前鉴别sMPLC和IM最可靠易行的方法之一。经过多年不断的发展，人们对于MPLC的病理学认识逐渐完善、细化。CHA及其衍生的其他方法是目前临床上用来判断MPLC和IM的主要方法，很多其他方法也常用CHA等作为参考标准，但是它们也有着自己的局限。比如它们的判断基于术后的病理，未能在术前对治疗提供准确的指导；需要获得足量的肿瘤样本；基于医生主观的判断等。这些不足在一定程度上造成了其在临床应用中的困扰。

## 2 影像学

影像学检查是肺癌筛查的“前哨兵”，几乎所有肺癌患者的首次发现均是通过影像学。胸部CT或正电子发射型计算机断层显像（positron emission computed tomography, PET）-CT是目前术前鉴别MLPC和IM的主要方法，临床医生常通过多个病灶的形态、肿瘤倍增时间等来综合判断，但是缺乏相对统一的标准，主观性较强。多灶性肺癌结节根据实性成分的多少在CT上常被分为以下4类：纯毛玻璃样结节/影（pure ground-glass nodules/opacities, pGGNs/GGOs）、毛玻璃样影为主的部分实性结节（GGO-predominant part-solid nodule, GGO-PSN）、实性成分为主的部分实性结节、纯实性结节^[12]^。结节内实性成分越多，往往提示更有侵袭性的病理类型^[13]^。表现为GGOs的肺内肿瘤被称为多发毛玻璃/鳞状（ground-glass/lepidic, GG/L）肺癌，GG/L肺癌主要包括微浸润腺（minimally invasive adenocarcinoma, MIA）、原位腺癌（adenocarcinoma *in situ*, AIS），通常被认为是独立的原发癌^[14]^。因此，有研究^[15]^认为至少有一个GGO的多灶性肺癌可以简单地认为是放射性MPLC。类似的结果也由Zhang等^[16]^的研究得出。但是两个GGO之间被证明也可以通过气腔播散（tumor spread through air space, STAS）的方式发生传播^[17]^，也就是说表现为GGOs的多发GG/L肺癌并不能完全被认为是MPLC。

标准化摄取值（standardized uptake value, SUV）被认为可以用来区分sMPLC和IM。SUV是PET-CT中常用的半定量指标，是指局部组织摄取的显像剂的放射性活度与全身平均注射活度的比值。SUV被广泛应用于肿瘤良恶性的判断和疗效评价。Dijkman等^[18]^计算患者肿瘤对之间的SUV差异值（difference between SUVs, ∆SUV），统计发现sMPLC患者的∆SUV明显高于转移患者。Liu等^[19]^研究中得出了同样的结果。但是这些结论仍需要更多证据。

简单的用单一的指标去鉴别MPLC和IM显然是不够全面和准确的，Suh等^[12]^将CT的放射学特征和PET-CT的SUV结合起来设计了一套新的算法去区分MPLC和IM。整个算法分为4个步骤：①肿瘤对是否至少有一个CT表现为GGO或GGO为主；②肿瘤对是否有毛刺征和支气管征；③肿瘤对的∆SUV是否超过两个等级；④是否存在N2/N3淋巴结转移或远处转移。如果满足以下条件之一，则将肿瘤对诊断sMPLC：任何肿瘤表现为具有纯GGO或GGO优势特征；两种肿瘤均带有毛刺征或支气管征；只有一个肿瘤带有毛刺或支气管征，并且肿瘤对具有超过两个等级的∆SUV；只有一个肿瘤带有毛刺或支气管征，且肿瘤对具有不超过两个等级的∆SUV，但是无N2/N3淋巴结转移或远处转移。这套算法得出来的结果与病理专家通过组织病理学审查结果高度一致。尽管这套算法仍缺乏足量的数据去支撑，但是其实现了在手术之前进行评估，对于更加精准的患者进行下一步的治疗指导有着重大的意义。

人工智能和深度学习的长足发展使得其在病理学、影像学、肿瘤学等医学领域有了诸多应用^[20, 21]^。这些技术的主要应用一直是肺结节的检测和分类。目前通过机器学习算法分析放射影像的特征来进行肺结节的检测、良恶性判断的水平达到甚至超过了人类专家^[22, 23]^，甚至可以用来预测表现为GGO的结节进一步恶化的可能性^[24]^。然而，人工智能应用于MPLC和IM鉴别鲜有报道。因此，基于机器学习的图像分析可能代表了一种在多灶性肺癌患者中识别MPLC和IM的新方法。

## 3 分子遗传学

随着现代分子生物技术的进步和发展，分子遗传学和分子生物学分析手段被更多地应用到多原发肺癌的鉴别诊断中。早在1995年Antakli等^[5]^对Martini和Melamed的标准进行补充时就增加了采用DNA倍体检测来区分MPLC和IM，但是由于技术及经济效益的制约，并未得到广泛应用。随着测序技术的不断进步，越来越多的证据用来支持MPLC的独立克隆起源^[25]^，这可能有助于IM和MPLC的鉴别诊断。目前，点突变、DNA甲基化、染色体重排等测序技术被报道可以用来区分MPLC和IM。

下一代测序技术（next generation sequencing, NGS）的蓬勃发展使得人们能够检测的基因数目不断扩大。最初人们只对少数的明星基因进行测序如*p53*、表皮生长因子受体（epidermal growth factor receptor, *EGFR*）、鼠类肉瘤病毒癌基因（kirsten ratsarcoma viral oncogene, KRAS）等来辅助诊断^[26, 27]^。这些研究往往基于一个或者两个热点突变基因来鉴别MPLC和IM。Girard等^[28]^提出，肿瘤对之间发生相同基因变化的可能性主要由基因突变频率决定，即如果多个肿瘤共享同一突变基因，如果这个基因的突变频率越低，多个肿瘤属于同一克隆起源的可能性越大，诊断价值愈大。相比之下，具有相同高频基因突变的患者在诊断他们的克隆来源是否相同时需要更加谨慎。随后Liu等^[29]^发现诊断为IM的同一患者不同肺肿瘤均可出现*EGFR* p.L858R突变和*EGFR*外显子19缺失突变等热点基因突变，但在其他基因位点具有明显的突变差异。此外，在不同组织病理学类型的多个肺肿瘤中可以检测到相同的热点基因突变。因此很难简单地通过几个热点突变就做出准确判断。Roepman等^[30]^指出对于类似于*EGFR*和*KRAS*的热点突变，多原发性肿瘤中偶然共享一个热点驱动基因突变的可能性很高。扩大检测基因数量，尤其是增加突变频率不高的基因数目可以进一步减少误差。Begg等^[31]^认为为了在统计学上达到更高的准确度，至少需要20个标记的基因突变位点来区别IM和MPL。一些对50个基因进行测序的研究^[32-36]^表明，如果多个肿瘤具有不同的驱动突变，那么它们是独立的；如果它们共享一个共同的驱动突变，那么它们是转移性的。这些基于较大基因面板的分子分类方法可以有效地提升准确性。但与此同时，这些研究也都表明尽管IM中的原发肿瘤和转移灶的基因突变一致率大于90%，而在考虑为MPLC的多发肺肿瘤时，仍有不低的概率基因突变一致。也就是说，即便提高了检测量，仍存在很大的误判可能。在这种情况下，分子遗传学和组织病理学结合成为更合理有效的选择。Mansuet-Lupo等^[37]^已经提出了一种针对MPLC的集成组织分子算法。当多个肿瘤共享一个频繁的热点突变（*EGFR*外显子19缺失或*EGFR* p.L858R或*KRAS* p.G12X）时，组织学算法是决定性的。此外，全基因组测序（whole genome sequencing, WGS）为克隆性评估提供了更全面的信息，但是由于其高成本和对肿瘤样品的要求，因此在临床环境中不切实际。总的来说，基于NGS技术的分子分类能够提供一种更加有效适用的方法。但是仍然存在一些问题，比如如何进一步提高准确性？尽管更大的基因面板测序甚至WGS可以在一定程度上解决这个问题，但并不能全面推广。其次当测序方法与病理组织方法矛盾时，谁是更准确的？这些问题需要进一步解决，但不能否定NGS技术在MPLC和IM鉴别方面的重大意义。

DNA甲基化（DNA methylation）指在DNA甲基化转移酶（DNA methyltransferase, Dnmt）的作用下将甲基选择性地添加到胞嘧啶上形成5-胞嘧啶的过程，为DNA化学修饰的一种形式，能够在不改变DNA序列的前提下，改变遗传表现。每种细胞类型都有独特的DNA甲基化谱，可作为追踪任何肿瘤细胞来源的工具^[38]^。Sano等^[39]^报告了一种分析十种基因启动子甲基化状态的系统，发现IM肿瘤对之间具有相同的甲基化谱，而MPLC却不同。它为鉴定多种肺癌的克隆关系提供了一种新工具。

染色体重排（chromosomal rearrangement）指断裂的染色体在重接过程中发生异常连接，导致染色体结构变异的现象。通常把染色体重排分为重复、缺失、倒位和易位四种类型。Murphy等^[40, 41]^用配对测序技术检测了体细胞断点连接，以确定肿瘤的谱系，MLPC肿瘤对中没有任何共同的基因组重排。而在IM患者中，在所有肿瘤对中均发现了共同的重排，这似乎可以用来鉴别MLPC和IM。

在蛋白质水平上，报道有了4种与癌症相关的蛋白质（p53、p16、p27和c-erbB2）在MPLC中差异表达。如果肿瘤之间4种蛋白质表达比率差异的总和大于90，则将诊断为MPLC^[25, 42]^。此外，微卫星不稳定性（microsatellite instability, MSI）和杂合性丢失（loss of heterozygosity, LOH）这两种现象都代表了细胞转化为恶性的过程中所发生的分子异常，将可用于区分多原发和转移^[43, 44]^，但是因为成本和技术原因难以推行。总而言之，分子分类在MLPC和IM的鉴别中有着独特的无可取代的作用，随着分子技术的不断的进步，我们相信越来越准确便携的方法会被开发及推广。

## 4 临床诊断方法概述

目前对于sMPLC和IM的鉴别诊断需要结合影像、病理和分子特征来进行全面评估。对于未手术或术前患者，在没有取得肿瘤组织样本时，可考虑使用影像综合评估的方法来进行判断（图 1）^[12]^。按照图 1所示流程逐步将多灶性肺癌分为sMPLC和IM。如果满足以下条件之一，则将肿瘤对诊断为sMPLC：肿瘤中至少有一个表现为纯GGO或GGO为主；肿瘤对均带有毛刺征或支气管征；只有一个肿瘤带有毛刺或支气管征，并且肿瘤对具有超过两个等级的∆SUV；只有一个肿瘤带有毛刺或支气管征，且肿瘤对具有不超过两个等级的∆SUV，但是无N2/N3淋巴结转移或远处转移。对于术后取得肿瘤组织样本的患者，若进行了NGS检测，则首先采用分子分类的算法（图 2）^[9, 37]^。当NGS未检测到突变，这时可以采用CHA进一步分析。当肿瘤对有突变且突变不同时，定义为sMLPC。当肿瘤对有两个及以上共同突变时，定义为IM。当肿瘤对共享一个常见的热点突变（*EGFR*外显子19缺失或*EGFR* p.L858R或*KRAS* p.G12X），需要进一步采用CHA的方法来评估。当肿瘤对共享一个其他的非热点突变包括*TP53*，可以直接定义为IM。若患者未进行NGS分析，则直接采用CHA来进行分类（图 3）。首先需要病理专家根据世界卫生组织（World Health Organization, WHO）2015年肺癌病理分类标准对患者进行病理分类评价。将百分比最高的成分定义为主要成分（如腺泡型、乳头型等）。然后采用图 3的流程来划分sMLPC和IM。满足以下条件之一，将肿瘤对诊断为sMLPC：肿瘤对至少有一个为AIS或MIA；肿瘤对具有不同的组织学类型；肿瘤对组织类型相同，主要成分不同，且肿瘤对在细胞学、基质特征上不同；肿瘤对组织类型相同，主要成分相同（主要成分为常见类型，如乳头状、腺泡型等），但是肿瘤对在细胞学、基质特征上不同。

**图 1:**
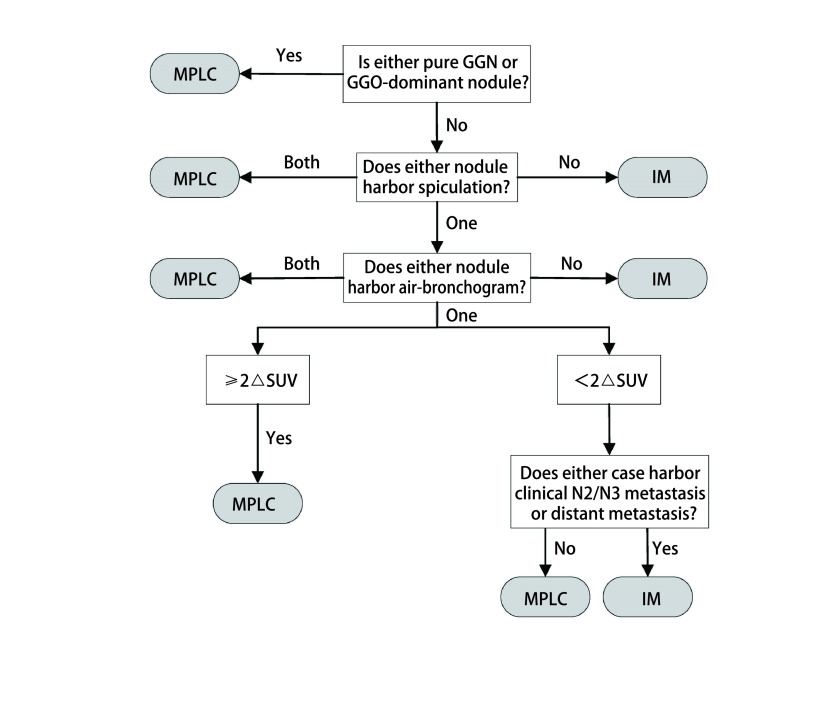
综合影像学评估流程图 Flowchart of radiological assessment. GGN: ground-glass nodules; GGO: ground-glass opacities; MPLC: multiple primary lung cancer; IM: intrapulmonary metastasis; SUV: standardized uptake value; ∆SUV: difference between SUVs.

**图 2:**
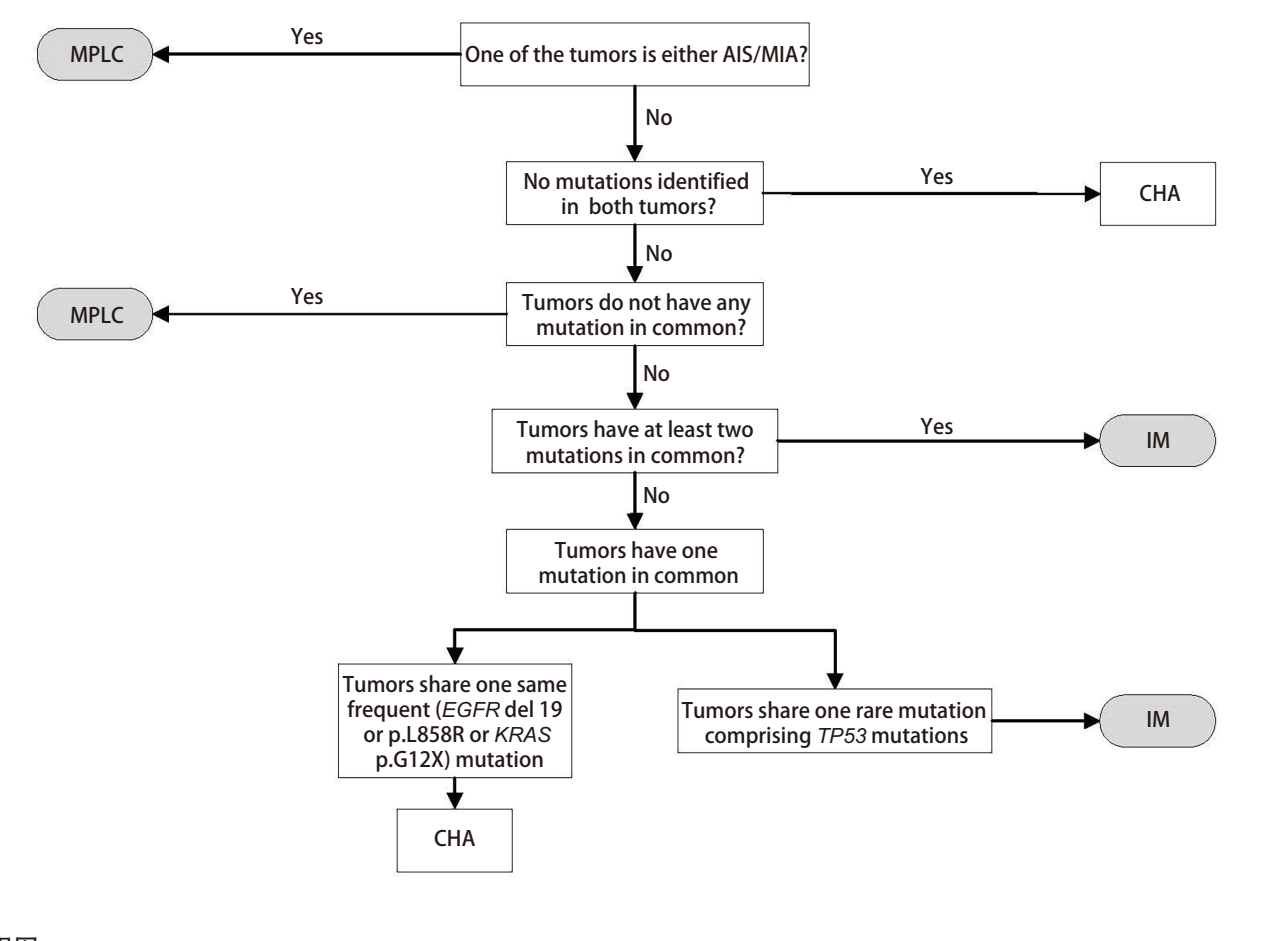
分子分类算法流程图 Flowchart of molecular algorithm. AIS: adenocarcinoma in situ; MIA: minimally invasive adenocarcinoma; CHA: comprehensive histologic assessment; EGFR: epidermal growth factor receptor; KRAS: kirsten rat sarcoma viral oncogene; TP53: tumor protein 53.

**图 3:**
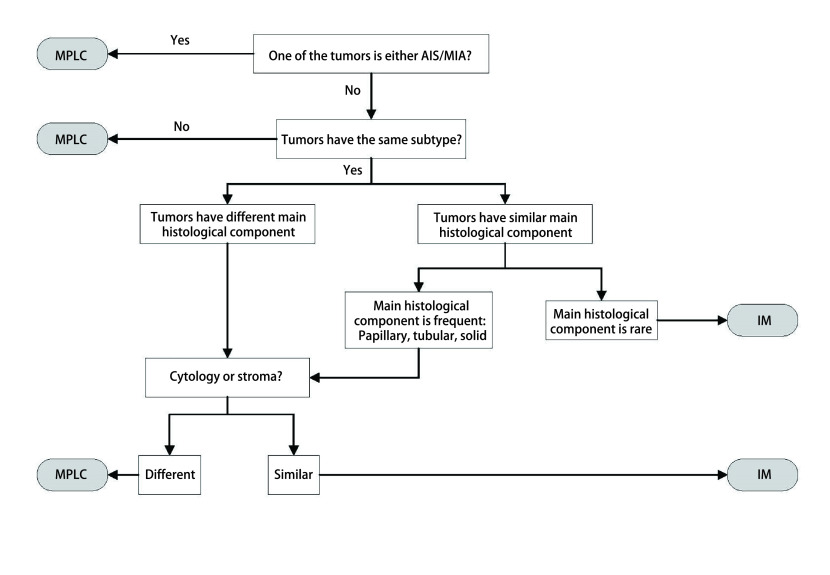
综合组织评估流程图 Flowchart of comprehensive histologic assessment

## 5 结论与展望

随着我国医疗检测水平的不断提高，尤其是影像学技术的进步，越来越多的多发肺癌被检测出来，而sMLPC和IM的鉴别仍是棘手问题。目前临床对于二者的区分更多依赖于医生的经验，而缺乏相对统一的标准。组织病理学、影像学、分子遗传学的发展为进一步提高判断能力提供了可能。组织学上，CHA及相关改进方法成为临床主流，但存在一定的局限。影像学方面，综合影像学分析会成为术前判断的利器。分子遗传学的进步为更加精准地鉴别sMPLC和IM提供了更多可能。可以大胆预测，未来对于MLPC和IM的鉴别绝不是单一学科可以解决的，多学科的联合会是最佳的出路。人工智能和机器学习的兴起，将会带来全新的解决思路，尤其在术前诊断方面有着不可替代的作用。测序的不断进步和深入，加之与组织病理学的联合，将会成为未来诊断sMLPC的新标准。
